# Dynamics of quasi-periodic, bifurcation, sensitivity and three-wave solutions for (*n* + 1)-dimensional generalized Kadomtsev-Petviashvili equation

**DOI:** 10.1371/journal.pone.0305094

**Published:** 2024-08-27

**Authors:** Muhammad Hamza Rafiq, Muhammad Bilal Riaz, Ghada Ali Basendwah, Nauman Raza, Muhammad Naveed Rafiq

**Affiliations:** 1 Department of Mathematics, University of the Punjab, Quaid-e-Azam Campus, Lahore, Pakistan; 2 Department of Mathematics and Statistics, The University of Lahore, Lahore, Pakistan; 3 IT4Innovations, VSB -Technical University of Ostrava, Ostrava, Czech Republic; 4 Department of Computer Science and Mathematics, Lebanese American University, Byblos, Lebanon; 5 Department of Mathematics, King Abdulaziz University, Jeddah, Saudi Arabia; 6 Department of Mathematics, Near East University, TRNC, Nicosia, Turkey; 7 School of Mathematics and Statistics, Central South University, Changsha, China; Julius-Maximilians-Universitat Wurzburg, GERMANY

## Abstract

This study endeavors to examine the dynamics of the generalized Kadomtsev-Petviashvili (gKP) equation in (*n* + 1) dimensions. Based on the comprehensive three-wave methodology and the Hirota’s bilinear technique, the gKP equation is meticulously examined. By means of symbolic computation, a number of three-wave solutions are derived. Applying the Lie symmetry approach to the governing equation enables the determination of symmetry reduction, which aids in the reduction of the dimensionality of the said equation. Using symmetry reduction, we obtain the second order differential equation. By means of applying symmetry reduction, the second order differential equation is derived. The second order differential equation undergoes Galilean transformation to obtain a system of first order differential equations. The present study presents an analysis of bifurcation and sensitivity for a given dynamical system. Additionally, when an external force impacts the underlying dynamic system, its behavior resembles quasi-periodic phenomena. The presence of quasi-periodic patterns are identified using chaos detecting tools. These findings represent a novel contribution to the studied equation and significantly advance our understanding of dynamics in nonlinear wave models.

## 1 Introduction

It is widely acknowledged within the scientific community that nonlinear evolution equations (NLEEs) play a crucial role in the investigation of nonlinear physical phenomena [1–5]. Contemporary research on multi-component integrable models has emerged as a significant advancement in the exploration of soliton equations [6–10]. In recent years, there has been a proliferation of powerful techniques for investigating the exact solutions of the NLEEs through the use of symbolic computation [11–15]. These methods include the modified simple equation method [16], the improved Bernoulli sub-equation method [17], Generalized logistic equation method [18], the Hirota direct approach [19, 20], multivariate bilinear neural network method [21], variable-coefficient symbolic computation approach [22], and the Lie symmetry approach [23], among others. The Hirota bilinear method, which was developed by Hirota, stands out as a powerful and effective approach for constructing exact solutions of NLEEs [24]. Once the bilinear form of a nonlinear equation is obtained by a dependent variable transformation, it becomes relatively effortless to obtain its multi-soliton solutions [24–26]. Furthermore, by utilizing the Lie symmetry analysis and dynamical system method, one can also derive the symmetries and exact explicit solutions of NLEEs [27, 28].

Complex system theory can be expounded as a mathematical branch that is devoted to scrutinizing dynamical systems that are characterized by a substantial number of variables [29]. The fundamental aim of this field is to extend the principles of dynamical system theory, which primarily concerns systems that are composed of a few variables. They have been widely used in various fields such as robotics, engineering structures, and science [30–32]. One illustrative example of a dynamical system is a framework utilized to encode trajectories from human demonstration, allowing for adaptability to changing environments and robustness to perturbations. Furthermore, the dynamical system of the hybrid electric vehicle incorporates front and back shaft drives, a battery, and a monitoring module which form an intricate system that requires the application of complex system theory to comprehend its dynamical behavior. In the present study, we aim to provide a comprehensive and in-depth analysis pertaining to the behavior of both the simple and perturbed dynamical systems of the nonlinear physical model that represents a physical phenomenon. Our discussion encompasses a range of topics such as the bifurcation analysis, sensitivity analysis, and the irregular and unpredictable behavior that may be observed within the desired dynamical system. Additionally, we shall employ a variety of efficient techniques and tools to facilitate our investigation.

Bifurcation refers to a topological and qualitative alteration that occurs in the phase space of a system when certain parameters undergo a slight modification beyond their critical thresholds [29]. Bifurcations assume significant functions in various tangible systems as a mode of switching. Such instances comprise the stimulation of neurons, the generation of patterns in morphogenesis (which will be expounded subsequently), the catastrophic shift of ecosystem states, and the storage of binary information in computer memory, among others. Seshasayanan et al. [33] investigate the primary bifurcations of a plane parallel flow with Kolmogorov forcing, including a new type called stationary drift bifurcation. Tang [34] delves into the exploration of bifurcation and dispersive soliton solutions within optical fibers utilizing the Schrödinger-Hirota equation.

Chaos refers to the persistent behavior of a nonlinear dynamic system that never follows fixed or repeating paths over time [29]. It may appear as a random variation, but it does occur within entirely deterministic and simple dynamic systems. Despite being subject to deterministic rules, chaotic systems manifest a remarkable susceptibility to initial conditions, rendering long-term prognoses and exact outcomes exceptionally arduous. In continuous time systems, chaos may arise in systems featuring no less than three independent dynamical variables, while simultaneously being nonlinear. These conditions must be met for the detection of chaos in continuous time systems. Whenever a system exhibits sensitivity to initial conditions, it is deemed chaotic. In essence, the definitive description of chaos states that a chaotic system is a deterministic one that displays behavior that appears random and unpredictable. The vast expanse of literature presents a plethora of methodologies for the identification of chaos [35]. These methodologies include time series analysis, three-dimensional phase portraits, Poincaré maps, power spectrum analysis, bifurcation diagrams, and Lyapunov exponents. These tools have demonstrated significant effectiveness in distinguishing the chaotic, quasi-periodic, and periodic patterns exhibited by physical models.

The Kadomtsev–Petviashvili (KP) equation, which was introduced by Kadomtsev and Petviashvili in 1970 as an analogue of the classic Korteweg-de Vries equation in two spatial dimensions, exhibits a high degree of efficacy in modeling nonlinear phenomena in various fields including fluid physics, plasma physics, Bose-Einstein condensates, optics, and beyond. The KP equation has garnered significant scholarly interest due to its physical and mathematical significance. Through the utilization of singularity manifold analysis, both Dorizzi et al. [36] and Wazwaz et al. [37] have demonstrated the integrability of the KP equation in instances of both constant and variable coefficients. Tian and Gao have successfully derived a variety of families of travelling wave solutions of the KP equation through their research efforts, as documented in their publication [38]. The lump solution, the lump and one stripe soliton, and resonance stripe soliton solutions for the KP equation were then subsequently constructed by utilizing the pioneering Hirota’s bilinear method [39]. Several research advancements have been accomplished in regards to the (3+1)-dimensional KP equation and its variants [5, 19, 40–48]. The KP system is a nonlinear partial differential equation that generalizes the Korteweg-de Vries (KdV) equation to two spatial dimensions. Its significance lies in its ability to model wave propagation across different physical domains, including shallow water waves and plasma physics. An in-depth comprehension of the outcomes linked to the KP system necessitates an analysis of solutions, stability, and the mathematical characteristics inherent in the system. This system represents a fertile research area within the realms of applied mathematics and theoretical physics, providing profound insights into the dynamics of nonlinear waves.

The proposed extended form of the KP equation in higher dimensions is presented as follows

$$\begin{array}{c} {\left(U_{t}+\alpha UU_{x_{1}}+\beta U_{x_{1}x_{1}x_{1}}\right)}_{x_{1}}+\gamma U_{x_{2}x_{2}}+\sum_{i=1}^{n}\;\sigma_{i}U_{x_{1}x_{i}}=0,\;n\geq 2, \end{array}$$ (1)

which is the generalized (*n* + 1)–dimensional KP equation. Ma et al. [49] have expanded on the KP equation in (*n* + 1)–dimensions by introducing a series of lump solutions produced from quadratic functions. This was followed by Chen et al. [50] who developed the multi-lump or lump-type solutions. Nevertheless, the extended KP equation is not integrable for *n* ≥ 3. If we take *x*_1_ = *x*, *x*_2_ = *y*, *α* = 6, *β* = 1, *γ* = 3*ρ*^2^, *σ*_1_ = *σ*_2_ = 0, then Eq (1) reads as

$$\begin{array}{c} {\left(U_{t}+6UU_{x}+U_{xxx}\right)}_{x}+3\rho^{2}U_{yy}=0,\;\rho^{2}=\pm 1, \end{array}$$ (2)

when the values of *ρ* are equal to *i* and 1, Eq (2) corresponds precisely to the KPI and KPII equations, respectively. The KP equation can be used to model water waves of long wavelength with weakly nonlinear restoring forces and frequency dispersion. The alteration in the sign of *ρ*^2^ is linked to the respective values of the gravitational and surface tension forces.

When the values of *x*_1_, *x*_2_, and *x*_3_ are assigned to *x*, *y*, and *z* respectively, Eq (2) can be simplified to the generalized KP equation in a (3+1)-dimensional space.

$$\begin{array}{c} {\left(U_{t}+\alpha UU_{x}+\beta U_{xxx}\right)}_{x}+\gamma U_{yy}+\sigma_{1}U_{xx}+\sigma_{2}U_{xy}+\sigma_{3}U_{xz}=0. \end{array}$$ (3)

It should be noted that a (3+1)-dimensional KP equation that was examined in [40] did not pass the test for integrability, as a result of the presence of a second-order dispersion term $U_{zz}$. In 2016, Wazwaz and El-Tantawy [51] discover the multiple solitons of model using the simplified Hirota’s direct method. They prove that the new model fails the Painleve integrability test although it gives multiple-soliton solutions. In 2017, Sonmezoglu et al. [52] investigated the exact solitary wave solutions of model. Also, Xu and Wazwaz [53] undertook a research endeavor on Eq (3) with the objective of investigating its integrability and localized solutions. Through the application of singularity manifold analysis and binary Bell polynomial method, it has been determined that the generalized KP equation of (*n* + 1)-dimensions contains *N*-soliton solutions, alongside the Painlevé property, Lax pair, Bäcklund transformation, and infinite conservation laws, thus establishing its complete integrability.

The aim of this research endeavor is to fabricate the three wave interactions and employ the Lie symmetry approach in order to identify the symmetry reductions, bifurcation analysis, sensitivity analysis and chaotic phenomena that arise in the intended dynamical system for Eq (3).

The subsequent sections of the paper are organized in the following order. In Section (2), the Hirota bilinear form that corresponds to the governing equation is presented. Section (3) serves as a tool for the construction of three-wave interactions, which are derived from the bilinear form presented in Section (2). In Section (4) and (5), we determine the symmetry reductions of the studied equation to obtain second order ordinary differential equation (ODE) using the Lie symmetry approach. Sec. (6) and (7) are used to study the bifurcation analysis and quasi-periodic behavior of the dynamical system. In Sec. (8) is used to perform sensitivity analysis using three different initial conditions. The main findings of the study are discussed in detail in Sec. (9). Sec. (10) provides the concluding remarks of study.

## 2 Hirota bilinear form of the governing equation

Consider the transformation defined as

$$\begin{array}{c} U\left(x,y,z,t\right)=\frac{12\beta }{\alpha }{\left(\text{ln}\;g\right)}_{xx}. \end{array}$$ (4)

Switching Eq (4) into Eq (3), we arrive at the following bilinear form as

$$\begin{array}{c} (D_{t}D_{x}+\beta D_{x}^{4}+\gamma D_{y}^{2}+\sigma_{1}D_{x}^{2}+\sigma_{2}D_{x}D_{y}+\sigma_{3}D_{x}D_{z})g\cdot g=0, \end{array}$$ (5)

where the Hirota bilinear operator is defined as

$$\begin{array}{c} D_{x}^{i}D_{y}^{j}g\cdot h=(\frac{\partial }{\partial x_{1}}-\frac{\partial }{\partial x_{2}}{)}^{i}(\frac{\partial }{\partial y_{1}}-\frac{\partial }{\partial y_{2}}{)}^{j}\times g\left(x_{1},y_{1}\right)h\left(x_{2},y_{2}\right){|}_{x_{1}=x_{2}=x,t_{1}=t_{2}=t}. \end{array}$$ (6)

Using the above formulas of Hirota operator, we acquire

$$\begin{array}{cc} & g_{xt}g-g_{t}g_{x}+\beta \left(g_{xxxx}g+3g_{xx}^{2}-4g_{x}g_{xxx}\right)+\gamma \left(g_{yy}g-g_{y}^{2}\right)+\sigma_{1}\left(g_{xx}g-g_{x}^{2}\right)\\ & +\sigma_{3}\left(g_{xy}g-g_{x}g_{y}\right)+\sigma_{4}\left(g_{xz}g-g_{x}g_{z}\right)=0. \end{array}$$ (7)

## 3 Three-wave solutions

The following test function is used to ensure that the solutions to Eq (3) has three-wave solutions

$$\begin{array}{c} g\left(x,y,z,t\right)=a_{1}e^{\rho_{1}}+e^{-\rho_{1}}+a_{2}\;\text{cos}\left(\rho_{2}\right)+a_{3}\;\text{sin}\left(\rho_{3}\right), \end{array}$$ (8)

where *ρ*_*i*_ = *j*_*i*_*x* + *k*_*i*_*y* + *l*_*i*_*z* + *m*_*i*_*t*, *i* = 1, 2, 3 and *j*_*i*_, *k*_*i*_, *l*_*i*_ and *m*_*i*_ are constants to be determined. By inserting Eq (8) for Eq (7) and equating like coefficients of different powers of $e^{\rho_{1}},e^{-\rho_{1}},\text{cos}\left(\rho_{2}\right),\text{sin}\left(\rho_{2}\right),\text{cos}\left(\rho_{3}\right)\text{sin}\left(\rho_{3}\right)$ and constant term to zero, we get:

$$\begin{array}{c} a_{1}=-\frac{\left(j_{2}^{2}-j_{3}^{2})(a_{2}^{2}j_{1}^{2}-a_{3}^{2}j_{1}^{2}+a_{2}^{2}j_{2}^{2}-a_{3}^{2}j_{3}^{2}\right)}{4(j_{1}^{2}+j_{2}^{2})(j_{1}^{2}+j_{3}^{2})},k_{1}=\frac{k_{2}j_{2}(j_{1}^{2}+j_{3}^{2})-k_{3}j_{3}(j_{1}^{2}+j_{2}^{2})}{j_{1}(j_{2}^{2}-j_{3}^{2})},\\ \gamma =\frac{3j_{1}^{2}\beta (j_{2}^{2}-j_{3}^{2})}{k_{2}j_{3}-k_{3}j_{2}},l_{1}=\frac{1}{\sigma_{4}j_{1}j_{2}^{2}(j_{2}^{2}-j_{3}^{2})}(j_{1}j_{2}^{2}m_{1}(j_{3}^{2}-j_{2}^{2})+j_{1}^{2}j_{1}^{4}\beta (3j_{2}^{2}-j_{1}^{2})\\ -2j_{1}^{2}j_{2}^{2}j_{3}^{2}\beta (j_{1}^{2}+3j_{2}^{2})+3j_{1}^{2}j_{3}^{4}\beta (j_{1}^{2}+j_{2}^{2})-\frac{3k_{2}^{2}\beta j_{1}^{4}(j_{2}^{2}-j_{3}^{2})}{{\left(k_{2}j_{3}-k_{3}j_{2}\right)}^{2}}(j_{2}^{2}+j_{3}^{2})\\ +\frac{6j_{1}^{2}j_{2}j_{3}(j_{2}^{2}-j_{3}^{2})}{{\left(k_{2}j_{3}-k_{3}j_{2}\right)}^{2}}(j_{1}^{2}k_{3}-j_{2}j_{3}k_{2}+j_{2}^{2}k_{3})+\sigma_{1}j_{1}^{2}j_{2}^{2}(j_{3}^{2}-j_{2}^{2})-k_{2}\sigma_{3}j_{2}^{3}(j_{1}^{2}+j_{3}^{2})+k_{3}\sigma_{3}j_{2}^{2}j_{3}(j_{1}^{2}+j_{2}^{2})),\\ l_{2}=\frac{1}{\sigma_{4}{\left(k_{2}j_{3}-k_{3}j_{2}\right)}^{2}}(j_{3}k_{2}m_{2}(2j_{2}k_{3}-j_{3}k_{2})-j_{2}^{2}k_{3}^{2}m_{2}+3j_{1}^{2}j_{2}k_{2}^{2}\beta (j_{3}^{2}-j_{2}^{2})+j_{2}j_{3}^{2}k_{2}^{2}\beta (j_{2}^{2}+j_{3}^{2})\\ +2j_{2}^{2}j_{3}k_{2}k_{3}\beta (3j_{1}^{2}-j_{2}^{2})-6j_{3}^{3}k_{2}k_{3}\beta (j_{1}^{2}+j_{2}^{2})+j_{2}^{3}k_{3}^{2}\beta (j_{2}^{2}-3j_{1}^{2})+3j_{2}j_{3}^{2}k_{3}^{2}\beta (j_{1}^{2}+j_{2})\\ +\sigma_{1}j_{2}j_{3}k_{2}(2j_{2}k_{3}-j_{3}k_{2})-\sigma_{1}j_{2}^{3}k_{3}^{2}+\sigma_{3}j_{3}k_{2}^{2}(2j_{2}k_{3}-j_{3}k_{2})-\sigma_{3}j_{2}^{2}k_{2}k_{3}^{2}),\\ l_{3}=\frac{1}{\sigma_{4}{\left(k_{2}j_{3}-k_{3}j_{2}\right)}^{2}}(j_{3}k_{2}m_{3}(2j_{2}k_{3}-j_{3}k_{2})-j_{2}^{2}k_{3}^{2}m_{3}+3j_{1}^{2}j_{3}k_{2}^{2}\beta (j_{2}^{2}-j_{3}^{2})+j_{3}^{3}k_{2}^{2}\beta (j_{3}^{2}+3j_{2}^{2})\\ +6j_{1}^{2}j_{2}k_{2}k_{3}\beta (j_{3}^{2}-j_{2}^{2})-2j_{2}j_{3}^{2}k_{2}k_{3}\beta (3j_{2}^{2}+j_{3}^{2})+3j_{2}^{2}j_{3}k_{3}^{2}\beta (j_{1}^{2}+j_{2}^{2})+j_{3}^{3}k_{3}^{2}\beta (j_{2}^{2}-3j_{1}^{2})\\ +\sigma_{1}j_{3}^{2}k_{2}(2j_{2}k_{3}-j_{3})-\sigma_{1}j_{2}^{2}j_{3}k_{3}^{2}+\sigma_{3}j_{3}k_{2}k_{3}(2k_{3}-j_{3}k_{2})-\sigma_{3}j_{2}^{2}k_{3}^{2}). \end{array}$$

We can obtain three-wave solution of the (*n* + 1)-dimensional generalized KP equation by using Eq (8) with above known parameter values as follows:

$$\begin{array}{cc} & U(x,y,z,t)=\\ & \frac{12\beta }{\alpha }(\frac{a_{1}j_{1}^{2}e^{\rho_{1}}+j_{1}^{2}e^{-\rho_{1}}-a_{2}j_{2}^{2}\;\text{cos}\;\left(j_{2}x+k_{2}y+l_{2}z+m_{2}t\right)-a_{3}j_{3}^{2}\;\text{sin}\;\left(j_{3}x+k_{3}y+l_{3}z+m_{3}t\right)}{a_{1}e^{\rho_{1}}+e^{-\rho_{1}}+a_{2}\;\text{cos}\;\left(j_{2}x+k_{2}y+l_{2}z+m_{2}t\right)+a_{3}\;\text{sin}\;\left(j_{3}x+k_{3}y+l_{3}z+m_{3}t\right)}\\ & -\frac{(a_{1}j_{1}e^{\rho_{1}}+j_{1}e^{-\rho_{1}}-a_{2}j_{2}\;\text{cos}\;\left(j_{2}x+k_{2}y+l_{2}z+m_{2}t\right)-a_{3}j_{3}\;\text{sin}\;\left(j_{3}x+k_{3}y+l_{3}z+m_{3}t\right){)}^{2}}{(a_{1}e^{\rho_{1}}+e^{-\rho_{1}}+a_{2}\;\text{cos}\;\left(j_{2}x+k_{2}y+l_{2}z+m_{2}t\right)+a_{3}\;\text{sin}\;\left(j_{3}x+k_{3}y+l_{3}z+m_{3}t\right){)}^{2}}). \end{array}$$ (9)

## 4 Lie symmetry analysis of Eq (3)

In this part, the aim is to identify the symmetry transformations of the studied equation. This is achieved by employing the well-known technique called Lie symmetry analysis, which was introduced by Marius Sophus Lie. Lie’s revolutionary ideas transformed the exploration of continuous symmetries and offered a potent approach to comprehending and resolving differential equations. Nowadays, Lie symmetry analysis persists as a dynamic field of study, discovering novel applications across diverse disciplines.

Let us examine the Lie group of infinitesimal transformations in (*x*, *y*, *z*, *t*, *q*), which is characterized by a single parameter.

$$\begin{array}{ccc} x^{*} & = & x+\varepsilon \rho^{1}\left(x,y,z,t,U\right)+O\left(\varepsilon^{2}\right),\\ y^{*} & = & y+\varepsilon \rho^{2}\left(x,y,z,t,U\right)+O\left(\varepsilon^{2}\right),\\ z^{*} & = & z+\varepsilon \rho^{3}\left(x,y,z,t,U\right)+O\left(\varepsilon^{2}\right),\\ t^{*} & = & t+\varepsilon \tau \left(x,y,z,t,U\right)+O\left(\varepsilon^{2}\right),\\ U^{*} & = & U+\varepsilon \zeta \left(x,y,z,t,U\right)+O\left(\varepsilon^{2}\right), \end{array}$$

with group parameter *ε* ≪ 1 and *ρ*^1^, *ρ*^2^, *ρ*^3^, *τ* and *ζ* are coefficient functions. The vector field which is associated with the previously mentioned Lie group can be described in the following way:

$$\begin{array}{cc} \text{V}=\rho^{1}\left(x,y,z,t,U\right)\frac{\partial }{\partial x}+\rho^{2}\left(x,y,z,t,U\right)\frac{\partial }{\partial y}+\rho^{3}\left(x,y,z,t,U\right)\frac{\partial }{\partial z} & \\ +\tau \left(x,y,z,t,U\right)\frac{\partial }{\partial t}+\zeta \left(x,y,z,t,U\right)\frac{\partial }{\partial q}, \end{array}$$ (10)

where the coefficient functions $\rho^{1}\left(x,y,z,t,U\right)$, $\rho^{2}\left(x,y,z,t,U\right)$, $\rho^{3}\left(x,y,z,t,U\right)$, τ(x,y,z,t,U) and ζ(x,y,z,t,U) are to be evaluated later. The invariance condition for Eq (4) with V becomes:

$$\begin{array}{c} Pr^{\left(4\right)}\text{V}({\left(U_{t}+\alpha UU_{x}+\beta U_{xxx}\right)}_{x}+\gamma U_{yy}+\sigma_{1}U_{xx}+\sigma_{2}U_{xy}+\sigma_{3}U_{xz}){|}_{Eq.(1)=0}=0, \end{array}$$ (11)

where $Pr^{\left(4\right)}\text{V}$ is a fourth prolongation of V and is defined as:

$$\begin{array}{c} Pr^{\left(4\right)}=U+\zeta^{tx}\frac{\partial }{\partial U_{tx}}+\zeta^{xx}\frac{\partial }{\partial U_{xx}}+\zeta^{yy}\frac{\partial }{\partial U_{yy}}+\zeta^{xy}\frac{\partial }{\partial U_{xy}}+\zeta^{xz}\frac{\partial }{\partial U_{xz}}+\zeta^{xxxx}\frac{\partial }{\partial U_{xxxx}}. \end{array}$$ (12)

By employing the prolongation defined in Eq (12) and conducting a coefficient comparison for the different derivatives of the dependent variable, we get a linear system of PDEs that is over-determined. Subsequently, after carrying out some computations, we get the set of determining PDEs. After solving the system of PDEs, we are able to successfully obtain the following set of symmetry transformations.

$$\begin{array}{c} \left\{ \begin{array}{c} {\text{V}}_{1}=\frac{\partial }{\partial x},{\text{V}}_{2}=\frac{\partial }{\partial y},{\text{V}}_{3}=\frac{\partial }{\partial z},{\text{V}}_{4}=\frac{\partial }{\partial t},\\ {\text{V}}_{5}=\frac{1}{2\gamma }\left(\sigma_{2}t-y\right)\frac{\partial }{\partial x}+\frac{\partial }{\partial y},{\text{V}}_{6}=F_{1}^{\prime }\left(t\right)\frac{\partial }{\partial u}+F_{1}\left(t\right)\frac{\partial }{\partial x},\\ {\text{V}}_{7}=U\frac{\partial }{\partial U}-\frac{3}{2}t\frac{\partial }{\partial t}-\frac{1}{4\gamma }\left(4\sigma_{1}\gamma t-{\sigma_{2}}^{2}t+\sigma_{2}y+2\gamma x\right)\frac{\partial }{\partial x},\\ {\text{V}}_{8}=F_{2}^{\prime }\left(t\right)y\frac{\partial }{\partial U}+\{F_{2}\left(t\right)\alpha y+\int \left(-F_{2}\left(t\right)\alpha \sigma_{2}\right)dt\}\frac{\partial }{\partial x}+(\int (-2\alpha \sigma_{2}F_{2}\left(t\right))dt)\frac{\partial }{\partial y},\\ {\text{V}}_{9}=-\frac{1}{2\gamma }\left(4\alpha F_{3}^{\prime }\left(t\right)\gamma U+F_{3}^{\prime \prime \prime }\left(t\right)y^{2}-2F_{3}^{\prime }\left(t\right)x\gamma \right)\frac{\partial }{\partial U}+3\alpha F_{3}^{\prime }\left(t\right)\frac{\partial }{\partial t}\\ +\frac{1}{2\gamma }\{-F_{3}^{\prime \prime }\left(t\right)\alpha y^{2}+F_{3}^{\prime }\left(t\right)\alpha \sigma_{2}y+2\alpha F_{3}^{\prime }\left(t\right)\gamma x+F_{3}\left(t\right)\alpha \sigma_{2}y+2(\int \frac{1}{2\gamma }\alpha (F_{3}^{\prime \prime }\left(t\right)\sigma_{2}y\\ -2F_{3}^{\prime \prime }\gamma x+4F_{3}^{\prime }\left(t\right)\sigma_{1}\gamma -F_{3}^{\prime }\left(t\right)\sigma^{2}-F_{3}^{\prime }\left(t\right)\sigma_{2}y+2F_{3}^{\prime }\left(t\right)\gamma x-F_{3}\left(t\right)\sigma^{2})dt)\gamma \}\frac{\partial }{\partial x}\\ +\left(2\alpha F_{3}^{\prime }\left(t\right)y+\int \left(-F_{3}\left(t\right)\alpha \sigma_{2}\right)dt\right)\frac{\partial }{\partial y}+3F_{3}\left(t\right)\alpha \sigma_{3}\frac{\partial }{\partial z}, \end{array}\right. \end{array}$$ (13)

where *F*_1_(*t*), *F*_2_(*t*) and *F*_3_(*t*) are free arbitrary functions. Symmetry transformations given in Eq (13) can be used to reduce the dimension of the considered equation. Therefore, in the next portion, we determine the reduced dimensions of the governing equation using computed symmetry transformations.

## 5 Symmetry reductions

As mentioned in the previous section, we have to determine the reduced dimensions of the governing equation using the symmetry transformations given in Eq (13). The reason behind to introduce this section is that we can obtain an ODE which is further used to investigate the qualitative analysis and chaotic structures of the considered equation.

**Reduction using subalgebra**: ${\text{V}}_{1}=\frac{\partial }{\partial x}$

We assume the characteristic equation for ${\text{V}}_{1}$ as

$$\begin{array}{c} \frac{dx}{1}=\frac{dy}{0}=\frac{dz}{0}=\frac{dt}{0}=\frac{dU}{0}. \end{array}$$ (14)

The desired solution for the characteristic Eq (14) is

$$\begin{array}{c} y=\xi_{1},\;z=\xi_{2},\;t=\xi_{3},\;U=W\left(\xi_{1},\xi_{2},\xi_{3}\right). \end{array}$$ (15)

Using the Eq (15) in Eq (3), we get the following result:

$$\begin{array}{c} W\left(\xi_{1},\xi_{2},\xi_{3}\right)=\xi_{1}F\left(\xi_{2},\xi_{3}\right)+G\left(\xi_{2},\xi_{3}\right), \end{array}$$ (16)

where $F(\xi_{2},\xi_{3})$ and $G(\xi_{2},\xi_{3})$ are arbitrary functions of *ξ*_2_ and *ξ*_3_.

**Reduction using subalgebra**: ${\text{V}}_{2}=\frac{\partial }{\partial y}$

We assume the characteristic equation for ${\text{V}}_{2}$ as

$$\begin{array}{c} \frac{dx}{0}=\frac{dy}{1}=\frac{dz}{0}=\frac{dt}{0}=\frac{dU}{0}. \end{array}$$ (17)

The desired solution for the characteristic Eq (17) is

$$\begin{array}{c} x=\xi_{1},\;z=\xi_{2},\;t=\xi_{3},\;U=W\left(\xi_{1},\xi_{2},\xi_{3}\right). \end{array}$$ (18)

Using the Eq (18) in Eq (3), we get the following PDE:

$$\begin{array}{c} W_{\xi_{3}}+\alpha WW_{\xi_{1}}+\beta W_{\xi_{1}\xi_{1}\xi_{1}}+\sigma_{1}W_{\xi_{1}}+\sigma_{3}W_{\xi_{2}}=0. \end{array}$$ (19)

Eq (19) has the following three translation symmetries:

$$\begin{array}{c} \{\eta_{1}=\frac{\partial }{\partial \xi_{1}},\;\eta_{2}=\frac{\partial }{\partial \xi_{2}},\;\eta_{3}=\frac{\partial }{\partial \xi_{3}}\}. \end{array}$$

Symmetry *η*_1_ + *η*_2_ + *η*_3_ yields the following invariants:

$$\begin{array}{c} \tau_{1}=\xi_{1}-\xi_{3},\;\tau_{2}=\xi_{2}-\xi_{3},\;W=\chi \left(\tau_{1},\tau_{1}\right). \end{array}$$ (20)

Substituting Eq (20) into Eq (19), we have

$$\begin{array}{c} \left(\sigma_{1}-1\right)\chi_{\tau_{1}}+\left(\sigma_{3}-1\right)\chi_{\tau_{2}}+\alpha \chi \chi_{\tau_{1}}+\beta \chi_{\tau_{1}\tau_{1}\tau_{1}}=0. \end{array}$$ (21)

The infinitesimal generators of Eq (21) are

$$\begin{array}{c} \mu_{\tau_{1}}=-\frac{1}{2}c_{1}\tau_{1}+\frac{\left(\alpha c_{2}-c_{1}\sigma_{1}+c_{1}\right)\tau_{2}}{\sigma_{3}-1}+c_{4},\;\mu_{\tau_{2}}=-\frac{3}{2}c_{1}\tau_{2}+c_{3},\;\mu_{\chi }=c_{1}\chi +c_{2}, \end{array}$$ (22)

with *c*_1_, *c*_2_, *c*_3_ and *c*_4_ arbitrary constants. Therefore, the Lagrange system of Eq (22) is

$$\begin{array}{c} \frac{d\tau_{1}}{-\frac{1}{2}c_{1}\tau_{1}+\frac{\left(\alpha c_{2}-c_{1}\sigma_{1}+c_{1}\right)\tau_{2}}{\sigma_{3}-1}+c_{4}}=\frac{d\tau_{2}}{-\frac{3}{2}c_{1}\tau_{2}+c_{3}}=\frac{d\chi }{c_{1}\chi +c_{2}}. \end{array}$$ (23)

The group invariant solution of Eq (23) for *c*_1_ ≠ 0 and *c*_2_ = *c*_3_ = *c*_4_ = 0 is

$$\begin{array}{c} \chi \left(\tau_{1},\tau_{2}\right)=[\frac{\phi (s)}{{\tau_{2}}^{2}}{]}^{\frac{1}{3}},\;s=\frac{\tau_{1}}{{\tau_{2}}^{\frac{1}{3}}}-\frac{\left(\sigma_{1}-1\right)}{\left(\sigma_{3}-1\right)}{\tau_{2}}^{\frac{2}{3}}. \end{array}$$ (24)

Inserting Eq (24) into Eq (21), we get

$$\begin{array}{c} \frac{1}{3}\alpha \phi^{\prime }\phi^{\frac{7}{3}}+\frac{10}{27}\beta {\left(\phi^{\prime }\right)}^{3}-\frac{2}{3}\phi^{\prime }\phi^{\prime \prime }+\frac{1}{3}\phi^{\prime \prime \prime }\phi^{2}-\phi^{2}\left(\sigma_{3}-1\right)[\frac{2}{3}\phi +\frac{s}{9}\phi^{\prime }]=0. \end{array}$$ (25)

**Reduction using subalgebra**: $\lambda_{1}{\text{V}}_{1}+\lambda_{2}{\text{V}}_{2}+\lambda_{3}{\text{V}}_{3}+{\text{V}}_{4}=\lambda_{1}\frac{\partial }{\partial x}+\lambda_{2}\frac{\partial }{\partial y}+\lambda_{3}\frac{\partial }{\partial z}+\frac{\partial }{\partial t}$.

We assume the characteristic equation for ${\text{V}}_{2}$ as

$$\begin{array}{c} \frac{dx}{\lambda_{1}}=\frac{dy}{\lambda_{2}}=\frac{dz}{\lambda_{3}}=\frac{dt}{1}=\frac{dU}{0}. \end{array}$$ (26)

The desired solution for the characteristic Eq (26) is

$$\begin{array}{c} x-\lambda_{1}t=\xi_{1},\;y-\lambda_{2}t=\xi_{2},\;z-\lambda_{3}t=\xi_{3},\;U\left(x,y,z,t\right)=W\left(\xi_{1},\xi_{2},\xi_{3}\right). \end{array}$$ (27)

Using the Eq (27) in Eq (3), we get the following PDE:

$$\begin{array}{c} \left(\sigma_{1}-\lambda_{1}\right)W_{\xi_{1}\xi_{1}}+\left(\sigma_{2}-\lambda_{2}\right)W_{\xi_{1}\xi_{2}}+\left(\sigma_{3}-\lambda_{3}\right)W_{\xi_{1}\xi_{3}}+\alpha {\left(WW_{\xi_{1}}\right)}_{\xi_{1}}\\ +\beta W_{\xi_{1}\xi_{1}\xi_{1}\xi_{1}}+\gamma W_{\xi_{2}\xi_{2}}=0. \end{array}$$ (28)

Eq (28) has the following three translation symmetries:

$$\begin{array}{c} \{\eta_{1}=\frac{\partial }{\partial \xi_{1}},\;\eta_{2}=\frac{\partial }{\partial \xi_{2}},\;\eta_{3}=\frac{\partial }{\partial \xi_{3}}\}. \end{array}$$

Symmetry *s*_1_*η*_1_ + *s*_2_*η*_2_ + *η*_3_ yields the following invariants:

$$\begin{array}{c} \tau_{1}=\xi_{1}-s_{1}\xi_{3},\;\tau_{2}=\xi_{2}-s_{2}\xi_{3},\;W\left(\xi_{1},\xi_{2},\xi_{3}\right)=\chi \left(\tau_{1},\tau_{1}\right). \end{array}$$ (29)

Substituting Eq (29) into Eq (28), we have

$$\begin{array}{c} \alpha {\left(\chi_{\tau_{1}}\right)}^{2}+\left(\sigma_{1}-\lambda_{1}-\left(\sigma_{3}-\lambda_{3}\right)s_{1}+\alpha \right)\chi_{\tau_{1}\tau_{1}}+\left(\sigma_{2}-\lambda_{2}-\left(\sigma_{3}-\lambda_{3}\right)s_{2}\right)\chi_{\tau_{1}\tau_{2}}\\ +\beta \chi_{\tau_{1}\tau_{1}\tau_{1}\tau_{1}}+\gamma \chi_{\tau_{2}\tau_{2}}=0. \end{array}$$ (30)

The infinitesimal generators of Eq (30) are

$$\begin{array}{c} \mu_{\tau_{1}}=c_{4}\;\mu_{\tau_{2}}=c_{3},\;\mu_{\chi }=c_{1}\tau_{2}+c_{2}, \end{array}$$ (31)

with *c*_1_, *c*_2_, *c*_3_ and *c*_4_ arbitrary constants. Therefore, the Lagrange system of Eq (31) is

$$\begin{array}{c} \frac{d\tau_{1}}{c_{4}}=\frac{d\tau_{2}}{c_{3}}=\frac{d\chi }{c_{1}\tau_{2}+c_{2}}. \end{array}$$ (32)

The group invariant solution of Eq (32) is

$$\begin{array}{c} \chi \left(\tau_{1},\tau_{2}\right)=\phi \left(s\right)+\frac{c_{1}}{2c_{3}}{\tau_{2}}^{2}+\frac{c_{2}}{c_{3}}\tau_{2},\;s=c_{3}\tau_{1}-c_{4}\tau_{2}. \end{array}$$ (33)

Inserting Eq (33) into Eq (30), we get

$$\begin{array}{c} \alpha {c_{3}}^{2}\left({\phi^{\prime }}^{2}+\phi^{\prime \prime }\right)+{c_{3}}^{2}\left(\sigma_{1}-\lambda_{1}-\left(\sigma_{3}-\lambda_{3}\right)s_{1}\right)\phi^{\prime \prime }-c_{3}c_{4}\left(\sigma_{2}-\lambda_{2}-\left(\sigma_{3}-\lambda_{3}\right)s_{2}\right)\phi^{\prime \prime }\\ +\beta {c_{3}}^{4}\phi^{\prime \prime \prime \prime }+\gamma (\frac{c_{1}}{c_{3}}+{c_{4}}^{2}\phi^{\prime \prime })=0. \end{array}$$ (34)

For convenience, we take *γ* = 0 in the above equation and it results into

$$\begin{array}{c} \alpha {c_{3}}^{2}\phi^{2}+2[{c_{3}}^{2}\left(\sigma_{1}-\lambda_{1}-\left(\sigma_{3}-\lambda_{3}\right)s_{1}\right)-c_{3}c_{4}\left(\sigma_{2}-\lambda_{2}-\left(\sigma_{3}-\lambda_{3}\right)s_{2}\right)]\phi +2\beta {c_{3}}^{4}\phi^{\prime \prime }=0. \end{array}$$ (35)

Eq (35) is seen to be of significant importance and has the potential to be used in further studies. Now, using the Galilean transformation to Eq (35), we obtain the subsequent dynamical system

$$\begin{array}{c} \left\{ \begin{array}{c} \frac{d\phi }{ds}=v,\\ \frac{dv}{ds}=d_{1}\phi -d_{2}\phi^{2}, \end{array}\right. \end{array}$$ (36)

where $d_{1}=\frac{{c_{3}}^{2}\left(\lambda_{1}-\sigma_{1}+\left(\sigma_{3}-\lambda_{3}\right)s_{1}\right)+c_{3}c_{4}\left(\sigma_{2}-\lambda_{2}-\left(\sigma_{3}-\lambda_{3}\right)s_{2}\right)}{\beta {c_{3}}^{4}}$ and $d_{2}=\frac{\alpha }{2\beta c_{3}^{2}}$.

## 6 Hamiltonian saddle-node bifurcation

Hamiltonian saddle-node bifurcations offer a key mechanism for exploring how stationary points evolve within a Hamiltonian system in response to parameter changes. Hamiltonian saddle-node bifurcations are of paramount importance in comprehending and forecasting the dynamics of various physical and engineering systems. These bifurcations are instrumental in elucidating the evolution of systems with varying parameters, providing essential insights into the stability, transitions and qualitative dynamics of Hamiltonian systems. For this, the Hamiltonian function corresponding to system (36) is given as:

$$\begin{array}{c} H\left(\phi ,v\right)=\frac{v^{2}}{2}-\frac{d_{1}}{2}\phi^{2}+\frac{d_{2}}{3}\phi^{3}=h, \end{array}$$ (37)

where *h* denotes the Hamiltonian constant. To find the equilibrium points of the dynamical system (36), we need to solve the following system of equations:

$$\begin{array}{c} \left\{ \begin{array}{c} v=0,\\ d_{1}\phi -d_{2}\phi^{2}=0. \end{array}\right. \end{array}$$ (38)

The equilibrium points of the system (36) are:

$$\begin{array}{c} P_{1}=\left(0,0\right),\;P_{2}=(\frac{d_{1}}{d_{2}},0). \end{array}$$

Furthermore, the Jacobian matrix of the linearized system (36) is computed and its determinant is given as

$$\begin{array}{c} H\left(\phi ,v\right)=|\begin{array}{cc} 0 & 1\\ d_{1}-2d_{2}\phi & 0 \end{array}|=2d_{2}\phi -d_{1}. \end{array}$$ (39)

As we know

I. If *H*(*ϕ*, *v*) < 0, then (*ϕ*, *v*) is a saddle point.

II. If *H*(*ϕ*, *v*) > 0, then (*ϕ*, *v*) is a center point.

III. If *H*(*ϕ*, *v*) = 0, then (*ϕ*, *v*) is a cuspidal point.

Based on the above points, the subsequent observations can be deduced concerning the real axis line *ϕ*:

*a*. (*ϕ*, 0) is a saddle point when $\phi <\frac{d_{1}}{2d_{2}}$.

*b*. (*ϕ*, 0) is a center point when $\phi >\frac{d_{1}}{2d_{2}}$.

*c*. (*ϕ*, 0) is a cuspidal point when $\phi =\frac{d_{1}}{2d_{2}}$.

The various outcomes that arise from varying the involved parameters are as follows:

**Case 1**: For *d*_1_ > 0 and *d*_2_ > 0

By choosing the suitable parameter values λ_1_ = 1, λ_2_ = *β* = 0.5 and *s*_1_ = *s*_2_ = *σ*_1_ = *σ*_2_ = *c*_3_ = *c*_4_ = *α* = 1. With reference to the present set of parameters, it can be observed that the two points of equilibrium are situated at coordinates (0, 0) and (1, 0), respectively. The visual depiction of their respective phase portraits can be found in Fig 2(a). These portraits show that (0, 0) is a saddle point, whereas (1, 0) is a centre point.

**Case 2**: For *d*_1_ < 0 and *d*_2_ > 0

By choosing the suitable parameter values λ_1_ = −0.5, λ_2_ = *β* = 0.5 and *s*_1_ = *s*_2_ = *σ*_1_ = *σ*_2_ = *c*_3_ = *c*_4_ = *α* = 1. With reference to the present set of parameters, it can be observed that the two points of equilibrium are situated at coordinates (0, 0) and (−2, 0), respectively. The visual depiction of their respective phase portraits can be found in Fig 2(b). These portraits show that (0, 0) is a center point, whereas (−2, 0) is a saddle point.

**Case 3**: For *d*_1_ > 0 and *d*_2_ < 0

By choosing the suitable parameter values λ_1_ = 1, λ_2_ = *β* = 0.5, *s*_1_ = *s*_2_ = *σ*_1_ = *σ*_2_ = *c*_3_ = *c*_4_ = 1 and *α* = −1. With reference to the present set of parameters, it can be observed that the two points of equilibrium are situated at coordinates (0, 0) and (−1, 0), respectively. The visual depiction of their respective phase portraits can be found in Fig 2(c). These portraits show that (0, 0) is a saddle point, whereas (−1, 0) is a centre point.

**Case 4**: For *d*_1_ < 0 and *d*_2_ < 0

By choosing the suitable parameter values λ_1_ = −0.5, λ_2_ = *β* = 0.5, *s*_1_ = *s*_2_ = *σ*_1_ = *σ*_2_ = *c*_3_ = *c*_4_ = 1 and *α* = −1. With reference to the present set of parameters, it can be observed that the two points of equilibrium are situated at coordinates (0, 0) and (2, 0), respectively. The visual depiction of their respective phase portraits can be found in Fig 2(d). These portraits show that (0, 0) is a center point, whereas (2, 0) is a saddle point.

## 7 Quasi-periodic behavior of perturbed autonomous system

To investigate the quasi-periodic and chaotic dynamics of the governing equation, we introduce a perturbation term cos(*ωs*) into the dynamical system (36). Consequently, the perturbed autonomous dynamical system of (36) with external periodic force can be written as follows:

$$\begin{array}{c} \left\{ \begin{array}{c} \frac{d\phi }{ds}=v,\\ \frac{dv}{ds}=d_{1}\phi -d_{2}\phi^{2}+g_{0}\text{cos}\left(\theta \right),\\ \frac{d\theta }{ds}=\omega , \end{array}\right. \end{array}$$ (40)

where *θ* = *ωs*. In system (40), the parameters denoted by *g*_0_ and *ω* correspond to the frequency and magnitude of the external force applied, respectively. The phenomenon of chaotic solutions in dynamical systems is a captivating subject that has received thorough investigation within the realm of nonlinear dynamics. The term “chaos” denotes a behavior distinguished by its sensitive reliance on initial conditions, its aperiodic and unpredictable trajectories, and the presence of a strange attractor within phase space. The identification and verification of chaotic structures in the perturbed dynamical system has been executed by means of chaos detecting tools including three dimensional phase portrait, Poincare map, time series analysis, Lyapunov exponents, as discussed in introduction section.

## 8 Sensitivity analysis

The importance of conducting sensitivity analysis on a dynamic system is rooted in its ability to provide valuable insights into how slight modifications in initial conditions or parameters can impact the behavior of the system. Such analysis is crucial in understanding the stability and predictability of intricate systems, and can assist in making informed decisions and formulating effective control strategies. In this section, we undertake a comprehensive study of the sensitivity of a dynamical system (36) at three distinct initial conditions, as illustrated in Fig 7. This analysis is performed under the suitable choice of parameter values and these are *d*_1_ = 2 and *d*_2_ = 3.5. It is observed that the dynamical system (36) is not highly sensitive; small variations in its initial conditions do not result in significant deviations from the path followed by the solution.

## 9 Results and discussion

**Fig 1:**
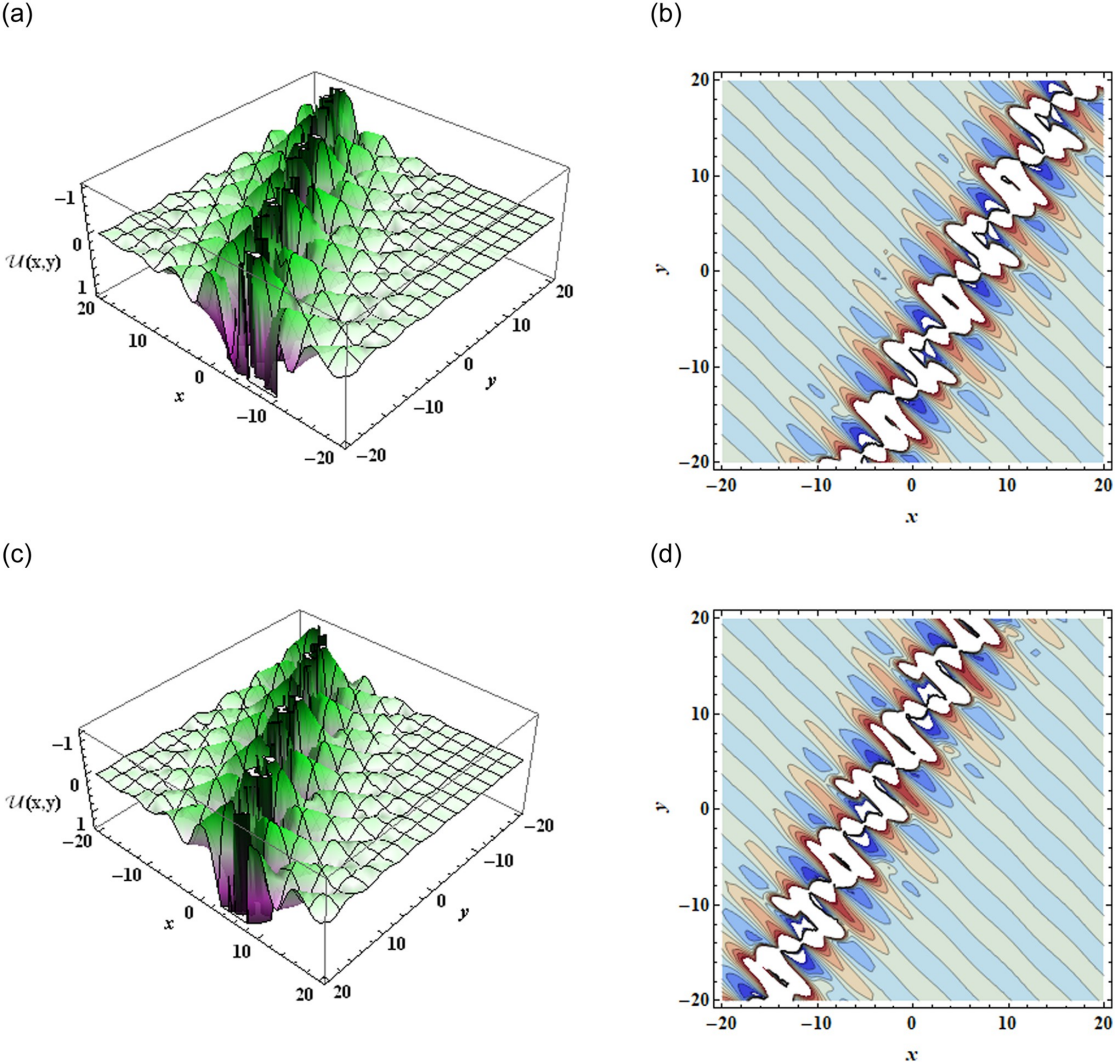
Graphical illustration of three-wave solution (9) in *x* − *y* plane when *a*_2_ = 0.1, *a*_3_ = 0.2, *j*_1_ = 0.16, *j*_2_ = 1, *j*_3_ = 0.04, *k*_2_ = *k*_3_ = *m*_1_ = *m*_2_ = *m*_3_ = 1, *α* = 0.21, *β* = −0.1, *σ*_1_ = 2.2, *σ*_3_ = 0.11, *σ*_4_ = −4.1.

**Fig 2:**
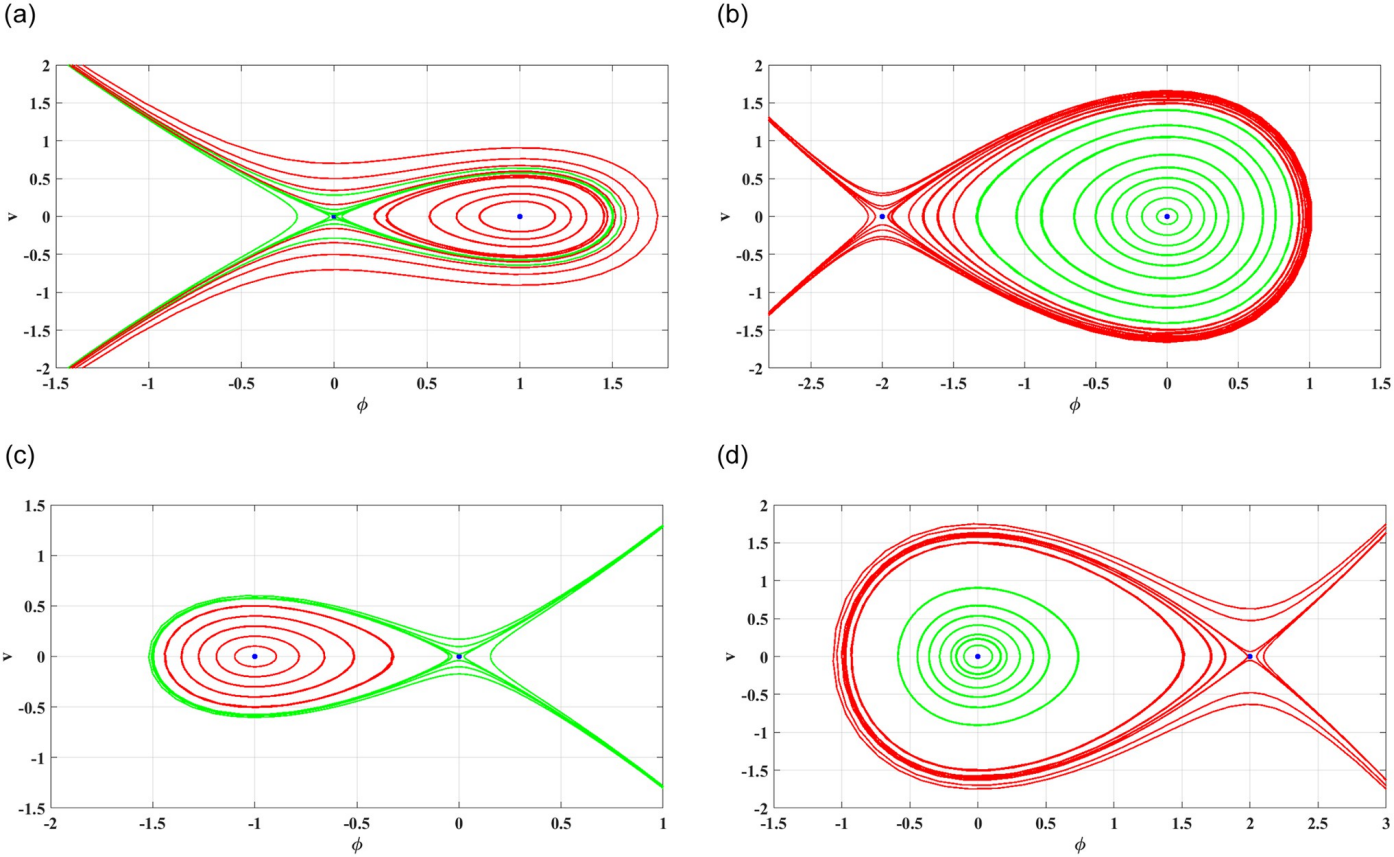
The phase portraits of Hamiltonian saddle-node bifurcation.

**Fig 3:**
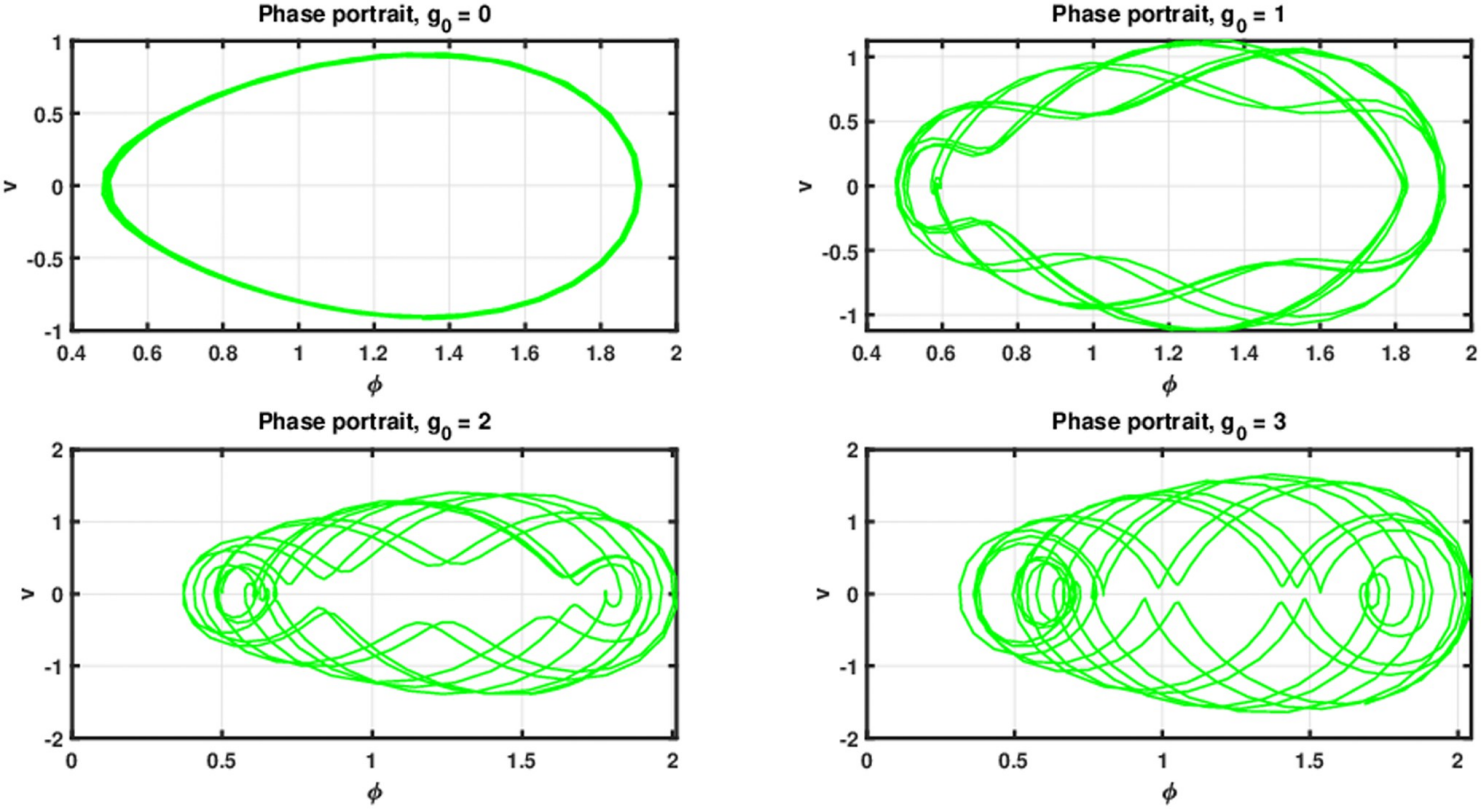
Phase portraits of periodic and quasi-periodic behavior in perturbed dynamical system (40) using initial condition (0.5, 0, 0.5) and *d*_1_ = 2, *d*_2_ = 1.5, *ω* = 4.5.

**Fig 4:**
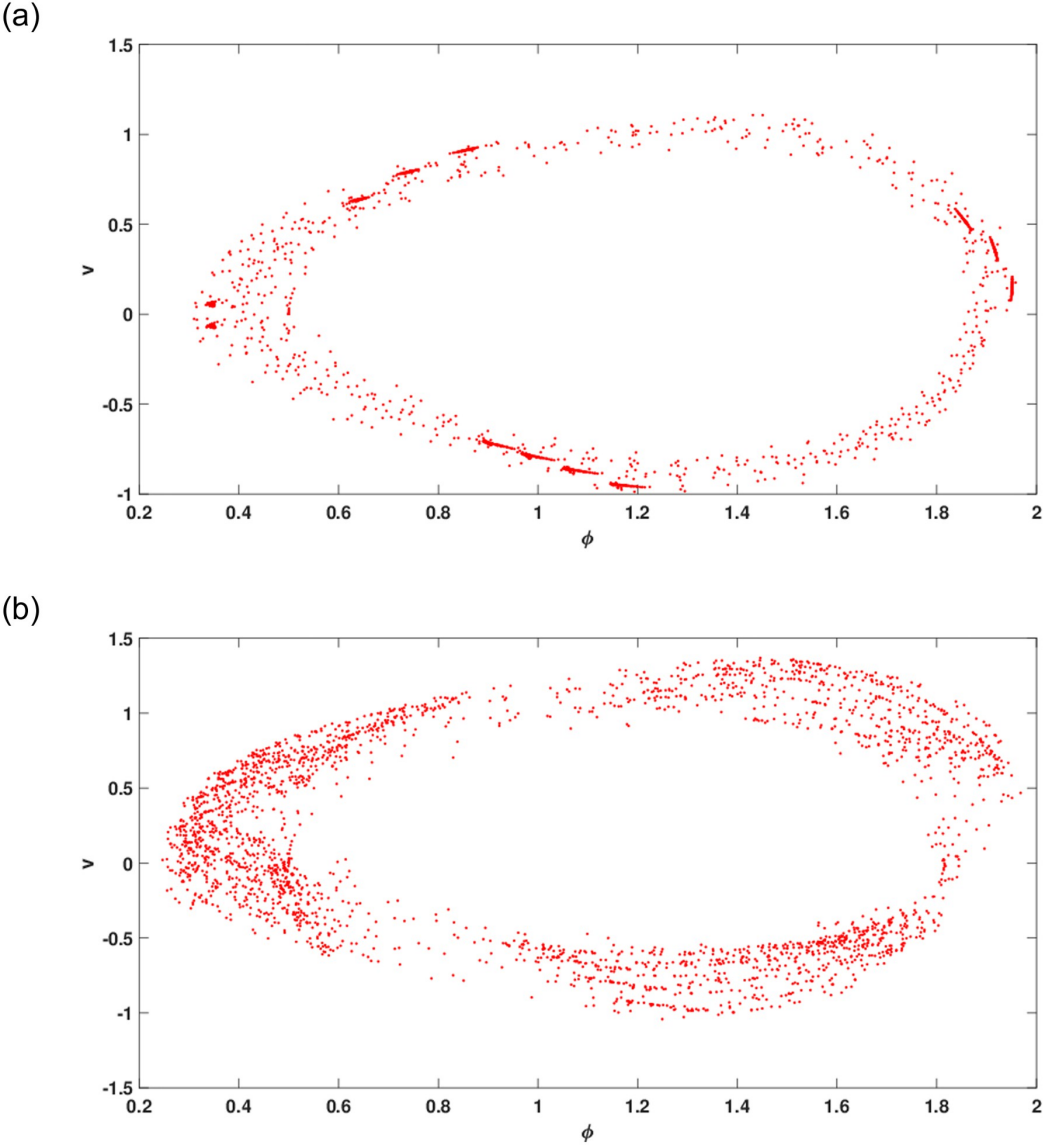
Poincare section for perturbed dynamical system (40) at initial condition (0.5, 0, 0.5) and *d*_1_ = 2, *d*_2_ = 1.5, *w* = 4.5.

**Fig 5:**
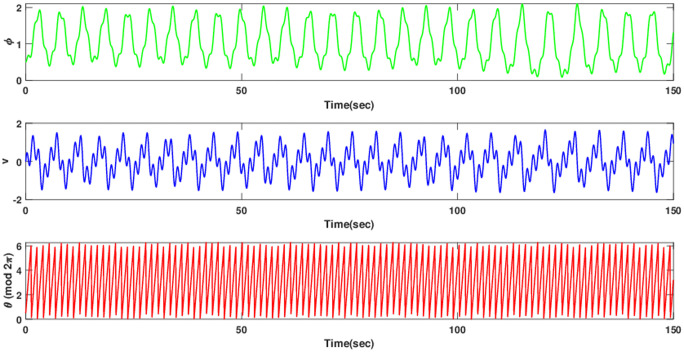
Time series analysis of perturbed dynamical system (40) using initial condition (0.5, 0, 0.5) and *d*_1_ = 2, *d*_2_ = 1.5, *ω* = 4.5.

**Fig 6:**
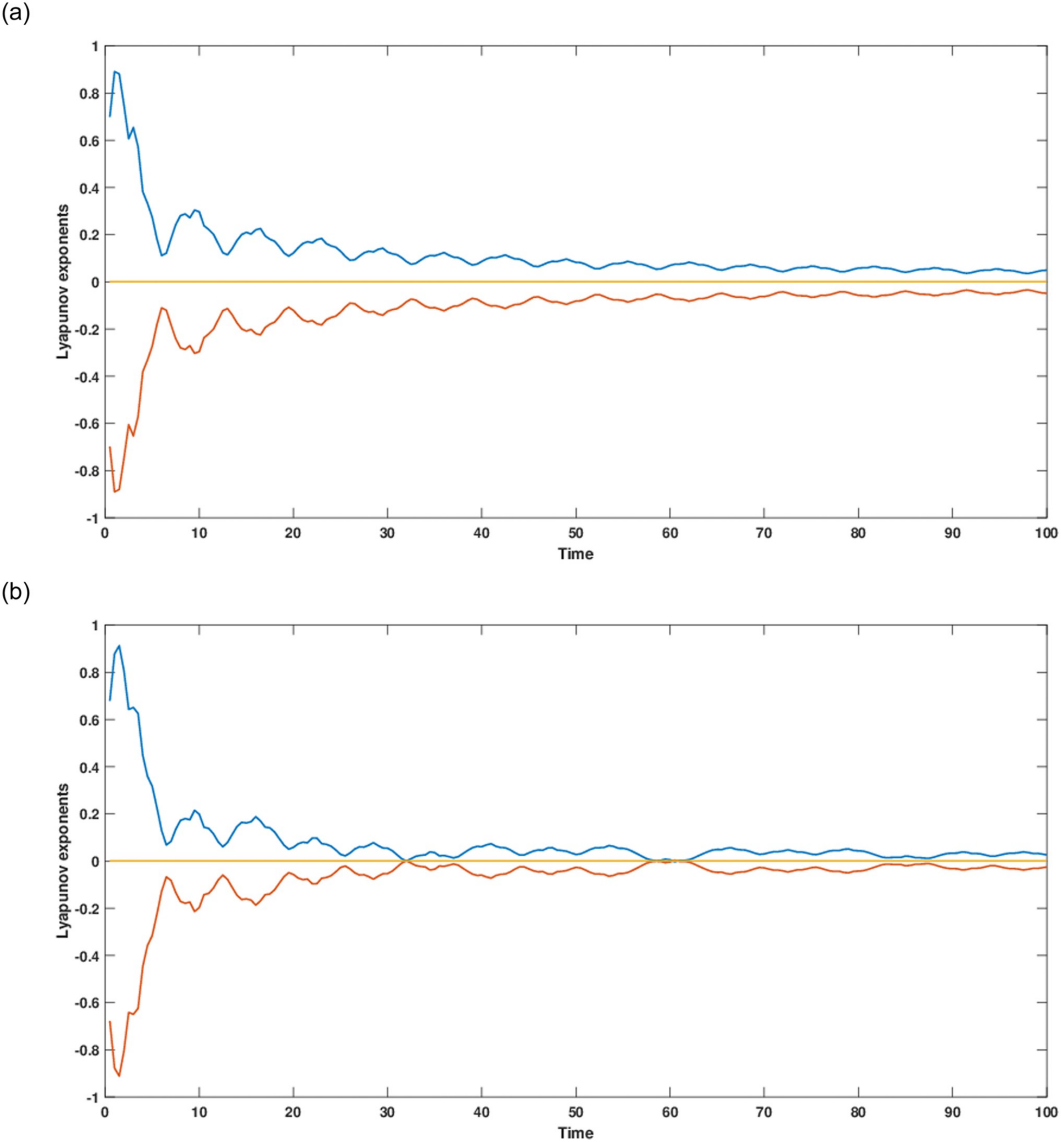
Dynamics of Lyapunov exponent for perturbed dynamical system (40) using time span [0, 100] and *d*_1_ = 2, *d*_2_ = 0.5, *w* = 4.5.

**Fig 7:**
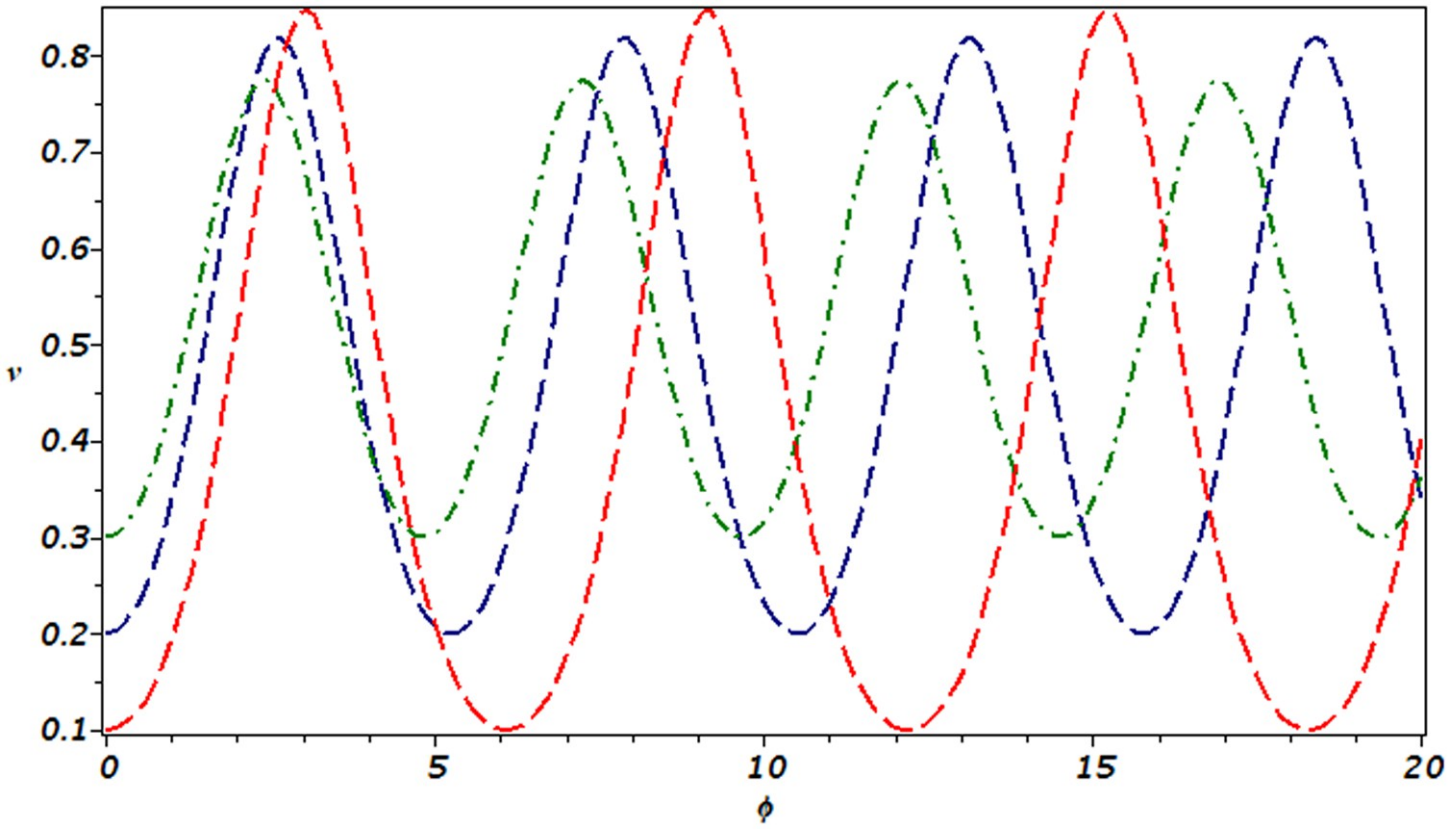
Sensitive analysis of dynamical system (36) for initial conditions (*ϕ*, *v*) = (0.1, 0) in red color, (*ϕ*, *v*) = (0.2, 0) in navy blue color and (*ϕ*, *v*) = (0.3, 0) in green color.

The current study involves development of new systems that explore useful findings in solitary waves theory. In Sec. (3), we discover the three-wave solutions using anstaz function and present them in 3D and density plots as shown in Fig 1. Sec. (6), signifies the dynamical observations of the planar dynamical system using bifurcation analysis. We show that how this dynamical system can be affected by its parameter values within the system, see Fig 2. Further, we introduce the perturbation term to show the quasi-periodic behavior in the nonlinear dynamical system using different chaos detecting tools available in the literature as shown in Figs 3–7. In Fig 3, the periodic and quasi-periodic behavior of the solution is observed using phase portrait tool. In Fig 4, we use Poincare section tool to identify the periodic and quasi-periodic behavior using *g*_0_ = 1 and *g*_0_ = 1.5. In Fig 5, time series analysis is performed for perturbed dynamical system at time span [0, 100] to observe the quasi-periodic behavior of solution. In Fig 6, we calculate the Lyapunov exponent values for a perturbed dynamical system at *g*_0_ = 0.5 and *g*_0_ = 2.5, given in Table 1. It also includes the sensitivity analysis of the planar dynamical system (36) at different initial values and observe the behavior of solutions as can be seen in Fig 7. The derived solutions could be very significant in elaborating physical aspects of real-world phenomena. The consequences of bifurcation and chaotic dynamics in dynamical systems encompass significant alterations in the qualitative behavior of the system, whereby minor perturbations in initial conditions can give rise to markedly diverse outcomes, rendering long-term forecasting challenging. Bifurcation may lead to the abrupt emergence of novel, stable states, whereas chaos introduces unreliability and susceptibility to initial conditions. These phenomena can have a substantial influence on domains such as meteorology, engineering, and financial markets, where grasping and handling the inherent unpredictability is essential for modeling and decision-making.

**Table 1:** Computation of Lyapunov exponent values for perturbed dynamical system (40) using time span [0, 100] and *d*_1_ = 2, *d*_2_ = 0.5, *w* = 4.5 (*a*) *g*_0_ = 0.5 and (*b*) *g*_0_ = 2.5.

Time	*g*_0_ = 0.5			*g*_0_ = 2.5		
	λ_1_	λ_3_	λ_3_	λ_1_	λ_2_	λ_3_
*t* = 2.5000	0.642366	-0.642366	0.000000	0.642366	-0.642366	0.000000
*t* = 5.0000	0.316703	-0.316703	0.000000	0.316703	-0.316703	0.000000
*t* = 7.5000	0.134473	-0.134473	0.000000	0.134473	-0.134473	0.000000
*t* = 10.0000	0.197762	-0.197762	0.000000	0.197762	-0.197762	0.000000
*t* = 12.5000	0.060052	-0.060052	0.000000	0.060052	-0.060052	0.000000
*t* = 15.0000	0.161077	-0.161077	0.000000	0.161077	-0.161077	0.000000
*t* = 17.5000	0.140172	-0.140172	0.000000	0.140172	-0.140172	0.000000
*t* = 20.0000	0.057535	-0.057535	0.000000	0.057535	-0.057535	0.000000
*t* = 22.5000	0.096235	-0.096235	0.000000	0.096235	-0.096235	0.000000
*t* = 25.0000	0.027808	-0.027808	0.000000	0.027808	-0.027808	0.000000
*t* = 27.5000	0.058539	-0.058539	0.000000	0.058539	-0.058539	0.000000
*t* = 30.0000	0.053647	-0.053647	0.000000	0.053647	-0.053647	0.000000
*t* = 32.5000	0.009483	-0.009483	0.000000	0.009483	-0.009483	0.000000
*t* = 35.0000	0.035255	-0.035255	0.000000	0.035255	-0.035255	0.000000
*t* = 37.5000	0.017915	-0.017915	0.000000	0.017915	-0.017915	0.000000
*t* = 40.0000	0.060383	-0.060383	0.000000	0.060383	-0.060383	0.000000
*t* = 42.5000	0.055447	-0.055447	0.000000	0.055447	-0.055447	0.000000
*t* = 45.0000	0.033905	-0.033905	0.000000	0.033905	-0.033905	0.000000
*t* = 47.5000	0.056083	-0.056083	0.000000	0.056083	-0.056083	0.000000
*t* = 50.0000	0.027328	-0.027328	0.000000	0.027328	-0.027328	0.000000
*t* = 52.5000	0.055697	-0.055697	0.000000	0.055697	-0.055697	0.000000
*t* = 55.0000	0.053303	-0.053303	0.000000	0.053303	-0.053303	0.000000
*t* = 57.5000	0.009598	-0.009598	0.000000	0.009598	-0.009598	0.000000
*t* = 60.0000	0.004691	-0.004691	0.000000	0.004691	-0.004691	0.000000
*t* = 62.5000	0.012152	-0.012152	0.000000	0.012152	-0.012152	0.000000
*t* = 65.0000	0.048519	-0.048519	0.000000	0.048519	-0.048519	0.000000
*t* = 67.5000	0.047087	-0.047087	0.000000	0.047087	-0.047087	0.000000
*t* = 70.0000	0.029630	-0.029630	0.000000	0.029630	-0.029630	0.000000
*t* = 72.5000	0.046885	-0.046885	0.000000	0.046885	-0.046885	0.000000
*t* = 75.0000	0.028708	-0.028708	0.000000	0.028708	-0.028708	0.000000
*t* = 77.5000	0.044456	-0.044456	0.000000	0.044456	-0.044456	0.000000
*t* = 80.0000	0.043088	-0.043088	0.000000	0.043088	-0.043088	0.000000
*t* = 82.5000	0.016313	-0.016313	0.000000	0.016313	-0.016313	0.000000
*t* = 85.0000	0.020507	-0.020507	0.000000	0.020507	-0.020507	0.000000
*t* = 87.5000	0.011103	-0.011103	0.000000	0.011103	-0.011103	0.000000
*t* = 90.0000	0.032755	-0.032755	0.000000	0.032755	-0.032755	0.000000
*t* = 92.5000	0.031545	-0.031545	0.000000	0.031545	-0.031545	0.000000
*t* = 95.0000	0.024393	-0.024393	0.000000	0.024393	-0.024393	0.000000
*t* = 97.5000	0.038501	-0.038501	0.000000	0.038501	-0.038501	0.000000
*t* = 100.0000	0.025755	-0.025755	0.000000	0.025755	-0.025755	0.000000

## 10 Conclusion

The work shows that the standard KP equation, and a variety of its extensions, give novel of integrable and non-integrable systems that lead to distinct physical structures. The work shows that this widely used model, that describes scientific features in plasma physics and fluid dynamics, explores many physical properties when extended to other versions. Firstly, we have applied the extended three-wave approach and Hirota’s bilinear method to obtain novel three-wave solutions. These solutions have been portrayed through figures to demonstrate an abundance of physical structures, as shown in Fig 1. Next, the Lie symmetry approach has been applied in order to elucidate the symmetry transformations that facilitate the reduction of equation dimensionality. Through utilization of the subalgebra of these transformations, a second-order differential equation has been successfully obtained. After that, we apply the Gallian transformation to the derived ordinary differential equation given in Eq (35). Sec. (6) offers an analysis of Hamiltonian saddle-node bifurcation and sensitivity of the dynamical system (36). In Fig 2, we have presented the phase portraits of Hamiltonian saddle-node bifurcation for different case of *d*_1_ and *d*_2_. To observe the quasi-periodic motion of the governing equation, we consider the perturbed dynamical system (40) involving perturbation term. When an external force is applied to the system (36), its behavior displays characteristics that bear similarity to those observed in chaotic phenomena. The identification of chaotic behavior in perturbed dynamical system is analyzed using phase portraits, Poincare sections, time series analysis and Lyapunov exponents as shown in Figs 3–6. Sensitivity analysis of the governing equation has done and it is observed that (see Fig 7) small variations in initial conditions of the dynamical system (36) do not result in significant deviations from the path followed by the solution. The derived solutions could be very significant in elaborating physical aspects of real-world phenomena. The final remarks presented in this article provide 325 additional insight into the evolutionary progression and physical mechanisms governing 326 solitons, thereby augmenting our comprehension of the nonlinear dynamics 327 characterizing solitons in optical communication systems.
